# Re–evaluation of the cost–effectiveness and effects of childhood rotavirus vaccination in Norway

**DOI:** 10.1371/journal.pone.0183306

**Published:** 2017-08-17

**Authors:** Christina Hansen Edwards, Birgitte Freiesleben de Blasio, Beatriz Valcárcel Salamanca, Elmira Flem

**Affiliations:** 1 Department of Infectious Disease Epidemiology and Modelling, Norwegian Institute of Public Health, Oslo, Norway; 2 Oslo Centre for Biostatistics and Epidemiology, Department of Biostatistics, Institute of Basic Medical Sciences, University of Oslo, Oslo, Norway; University of Groningen, NETHERLANDS

## Abstract

**Background:**

Rotavirus vaccination was included into the Norwegian childhood immunisation programme in 2014. Before implementation, rotavirus vaccination was found to be cost–effective from a societal perspective, but not from a healthcare perspective. Since introduction, new data on the incidence and economic effects of rotavirus disease have become available. We assessed early epidemiological effects of the rotavirus vaccination programme and re–evaluated its cost–effectiveness in Norway for the years 2015–2019.

**Methods:**

Using a dynamic transmission model, we compared the epidemiological effects of the ongoing two–dose vaccination programme with Rotarix^®^, and a hypothetical 3–dose programme with RotaTeq^®^ with no vaccination. A baseline cost of € 54 per fully vaccinated child was used. Cost–effectiveness was computed from a healthcare and societal perspective, using a decision analytical model. Data on healthcare use and costs, productivity losses and health utilities were based on published and own estimates. Uncertainty was accounted for in one–way, multi–way, and probabilistic sensitivity analyses.

**Results:**

During 2015–2019, 114,658 home care cases, 34,571 primary care cases, 7,381 severe cases, and 2 deaths associated with rotavirus disease were avoided due to vaccination. Under baseline assumptions vaccination was cost–effective from a healthcare perspective with a cost per QALY of € 47,447 for Rotarix^®^ and € 52,709 for RotaTeq^®^. The break–even price was € 70 for Rotarix^®^ and € 67 for RotaTeq^®^. Vaccination was cost–saving from the societal perspective, and also from a healthcare perspective for vaccine prices below € 25 and € 22 per vaccinated child for Rotarix^®^ and RotaTeq^®^, respectively.

**Conclusion:**

Ongoing childhood rotavirus vaccination in Norway has reduced the rotavirus disease burden substantially, and is cost–effective compared with no vaccination.

## Introduction

Rotavirus gastroenteritis (RVGE) leads to a considerable health and economic burden in unvaccinated populations [1]. In developing countries, mortality from rotavirus is common, while in high–income countries rotavirus–associated deaths are rare. [2, 3] The disease is largely preventable through vaccination [4], and in recent years, rotavirus vaccines have been introduced in vaccination programmes of 81 countries worldwide; eight of which are within the European Union. [5] Many studies clearly illustrate the health benefits of rotavirus vaccination, but whether these benefits outweigh the costs of vaccination is country–specific [6].

In Norway, the rotavirus vaccine was included in the national childhood immunisation programme in 2014. Two rotavirus vaccines are currently licenced for use in Norway: Rotarix^®^ (GlaxoSmithKline Biologicals, Rixensart, Belgium) and RotaTeq^®^ (Merck & Co., Inc., Whitehouse Station, N.J., U.S.A.). Rotarix^®^ is a human G1 monovalent vaccine, requiring two separate doses per child, while RotaTeq^®^ is a bovine–human reassortant pentavalent vaccine requiring three separate doses per child. As a part of the Norwegian childhood vaccination programme, Rotarix^®^ is offered free of charge to all children born on or later than 1 September 2014.

Our study is the first to describe the early effects of the ongoing rotavirus vaccination programme, and is the second study to evaluate the cost–effectiveness of rotavirus vaccination in the Norwegian setting. The previous health economic evaluation of universal childhood rotavirus vaccination concluded that vaccination would be cost–effective from a societal perspective, but not from a healthcare perspective [7], but this study was limited by the availability of data. The incidence of hospitalisation due to RVGE was estimated at three cases per 1000 children below 5 years of age [8], while the incidence of primary care consultations and homecare disease episodes was derived from the hospitalisation estimates. Since 2009, new and improved studies have assessed the health [9] and economic [10] burden of rotavirus in the primary and secondary healthcare sectors in Norway. These studies reported a higher incidence of rotavirus–associated primary care consultations (30.6 per 1000 children) and hospitalisations (6.3 per 1000 children) [9]. Rotavirus–associated mortality was estimated at 0.17 deaths per 100,000 children under 5 years of age [9]. These recent data suggest that the disease burden of RVGE was underestimated in the previous economic evaluation; therefore a re–evaluation is warranted. Since the vaccination programme has already been initiated, we were able to use new data on the burden of disease after vaccine introduction [9, 11]. We also applied new data concerning workdays lost among caregivers of hospitalized rotavirus cases to better estimate productivity losses [12].

In this study we combine data from the pre–and post– vaccination periods to estimate the initial epidemiological effects of the ongoing rotavirus vaccination programme in Norway, and re–evaluate the cost–effectiveness of the vaccination programme compared with no vaccination from the healthcare and societal perspectives.

## Materials and methods

### Study setting

Norway is located in Scandinavia and has a population of 5.2 million inhabitants [13]. The Norwegian healthcare system is mainly financed by the public sector and provides universal coverage to all residents. The childhood vaccination programme is included in this scheme, and all children are eligible to receive programme vaccines free of charge. Some private healthcare services are available, but the majority of hospitals are public. The healthcare system includes a primary and a secondary sector. Primary care has a gatekeeping role to secondary care. The primary care sector is organized at the municipal level, whereas secondary care is administered at the regional level. Norwegian parents have a right to 10 workdays of leave each, with full income compensation, to care for their ill children under the age of 12 years. Parents of multiple children and parents with single custody are entitled to additional days of leave. [14]

### Mathematical model

An extended version of a previously published [15–17] dynamic rotavirus model was adapted to the Norwegian setting. The model has an M–SEIRS (Maternal immunity, Susceptible, Exposed, Infected, Recovered, Susceptible) structure and includes separate modelling of the first, second, third and later rotavirus infections. We stratified the population into 74 age groups in the following way: <5 years (monthly age groups), 5–69 years (5-yearly age groups), and ≥70 years. Social mixing in the model was based on physical contact data from Finland collected as a part of the POLYMOD study on social contact patterns [18]. We fitted our model using weekly hospital sentinel data from March 2006 to February 2008 and from January 2014 to February 2015; 417 children aged <5 years of age with laboratory confirmed rotavirus infection were included. Vaccination began in mid-October 2014 and we assumed that vaccine-related changes in the rotavirus epidemiology before February 2015 were negligible. Due to the limited and time-interrupted data, we collapsed the sentinel data into a single year based on the weekly incidence of rotavirus prior to model fitting (S1 Fig). In the model fitting procedure we applied a burn–in period of 40 years to let the model reach a steady state using historic and projected data on births, mortality, and net immigration rates from 1970–2019 [13]. We estimated the average transmission rate by maximizing the likelihood under the assumption that the count of laboratory confirmed hospitalization events in each age group <5 years were multinomial distributed, as detailed by de Blasio et al.[16]. The model fit was repeated for all possible combinations of: 1) two different values for the infectivity of later infections; 2) reduced mixing in children <12 months, corresponding to the duration of the maternity leave in Norway; 3) reduced hospitalization rate for children >2.5 years with severe RVGE compared to younger children. In total, eight different models were tested. The final model included one parameter representing the relative reduced mixing among children under 12 months of age, and one parameter representing a reduced reporting rate in children above 2.5 years of age, which improved the maximum likelihood estimation model fit (S1 Table). Seasonality was incorporated in the model by including a sinusoidal forcing term to the transmission rate with a period of one year. Because the weekly sentinel observations were few, we fitted the amplitude and phase of the seasonal forcing separately and after the primary model selection.

Infections were classified as mild, severe or asymptomatic in accordance with Velázquez et al. [19], and the duration of immunity following natural infection was assumed to be one year[15, 20–22]. The susceptibility and infectivity was assumed to be reduced during the second and later infections relative to the first infection [19], (Table 1). The majority of rotavirus cases classified as severe were all assumed to require primary healthcare consultations [12]; the mean proportion was assumed to be 0.975 (range 0.95–1.0). Hospitalisations and deaths among children with severe infections were calibrated to recent Norwegian national estimates published by Bruun et al. [9]. Mild cases were assumed to be cared for at home without medical attention.

**Table 1:** Epidemiological model parameters.

Parameter	Value	Reference
**Demographic**		
Birth cohorts (1975–2019)^a^	Range (49 937–68 762)	[13]
Infant mortality (<1 year) per 1000 (1975–2019)^a^	Range (2.33–10.79)	[13]
Mortality 1–4 years per 1000 (1975–2019)^a^	Range (0.67–0.12)	[13]
Mortality 5–69 years per 1000 (1975–2019)^a^	Range (2.20–3.92)	[13]
Mortality 70+years per 1000 (1975–2019)^a^	Range (56.30–68.33)	[13]
Net immigration (1975–2019)^a^	Range (1 453–47 343)	[13]
Population January 1, 1975	3 972 990	[13]
**Natural history**		
Mean duration of maternal immunity (days)	102	[28, 29]
Mean duration of latency period (days)	0.5	[16, 17]
Mean duration of infectious period (1st; 2nd; 3rd and later) (days)	7; 3.5; 1.75	[30, 31]
Relative infectivity (1st; 2nd; 3rd and later)	1; 0.5; 0.2	Author assumption
Relative susceptibility (1st; 2nd; 3rd and later)	1; 0.62; 0.40	[19]
Mean duration of natural immunity (years)	1	[15, 20–22]
Proportion with RVGE/(severe RVGE) (1st; 2nd; 3rd and later)	0.47; 0.25; 0.24	[19]
Proportion severe RVGE (1st; 2nd; 3rd and later)	0.13; 0.04; 0	[19]
**Vaccination**		
Proportion with RVGE (among children with no previous infection)^b^	Uniform random (0.225–0.375)	[26]
Proportion severe RVGE (among children with no previous infection)^b^	Uniform random (0.01–0.04)	[26, 27]
Mean duration of vaccine–induced immunity (years)	2	[23, 25, 27, 32]
Coverage (max after introduction)	0.947 first dose (baseline); 0.877 second dose	[11]
**Healthcare and deaths in children under five years of age**		
Proportion of severe RVGE seeking primary healthcare	Uniform random (0.95–1.0)	Author assumption
Calibration of inpatient hospital contacts (2014)	Random Norm (1226, 86.5)	[9]
Calibration of outpatient hospital contacts (2014)	Random Norm (695, 28.3)	[9]
Calibration of fatal inpatient hospital contacts (2014)	Random Norm (4.218e–4, 0.35e–4)	[9]
**Fitted parameter values**		
Transmission parameter (β0)	0.48312	
Seasonal forcing (β1)^c^	0.0433	
Phase angle (φ)^c^	0.6191	
Relative mixing, children <12 months (mix_1y)	0.4803	
Relative reporting rate children >2.5 years (rep2,5y)	0.4629	

^a)^ Only minimum and maximum values of the demographic parameters are listed. All simulations were performed using the same set of demographic parameters.

^b)^ Proportions estimated to provide a vaccine efficacy of 0.93 (95% CI 0.87–0.98) against severe disease and 0.60 (95% CI 0.51–0.70) against mild disease. Vaccine efficacy calculated for children with no previous natural infection.

^c)^ Transmission rate modelled as: β0 (1+β1 (sin 2πt/ (365+φ))).

We simulated the impact of the national vaccination programme starting on 14 October 2014 until the end of 2019. In total, 10,000 simulations were run using the fitted model with random values for primary care consultations, hospital inpatient and outpatient contacts, and deaths; stochastic simulation was implemented to estimate healthcare encounters. Results were compared with the models’ projections without vaccination for a five–year period from 1 January 2015 until the end of 2019.

Vaccination was modelled as a two–dose regimen with Rotarix^®^ in accordance with evidence from clinical trials suggesting that vaccine-induced protection lasts for more than one year [23, 24], we assumed that the duration of vaccine induced protection was on average 2 years. In the base-case we assumed that the vaccine provided protection to all children after they received the first dose and that protection was achieved was at two months of life [25]. The proportion of severe symptomatic and symptomatic infections in children (with no prior infection) was assumed random with mean values of 0.025 and 0.300, respectively. These numbers were estimated to target vaccine efficacy values consistent with current estimates from high-income countries; the mean effectiveness against severe and symptomatic infection were 0.93 and 0.60, respectively [26, 27]. The vaccine was assumed to have an equal effect on susceptibility and infectivity as that of a natural infection [21] (Table 1).The effect of the second dose was not explicitly modelled. A logistic growth curve model was used to estimate rotavirus vaccination coverage during the first five years after the programme start. The model was simulated using real–world vaccination coverage rates observed in Norway during September 2014–March 2016 [11]. Coverage estimates with 95% confidence intervals were predicted by the model for the remaining study period of April 2016–December 2019.

### Cost–effectiveness study

The economic evaluation was based on the number of rotavirus inpatient and outpatient hospital cases, primary care consultations, and deaths estimated by the dynamic model. We evaluated the costs and effects of vaccination and no vaccination for the period 2015–2019. Because the phasing-in period in 2015 is relatively ineffective, and unlikely to be representative of coming years, we conducted a separate analysis for the period 2016–2019. For each vaccination strategy, we computed healthcare costs, productivity losses due to work absences, and health–related quality of life detriments. In the vaccination strategy, we added the costs of the vaccination programme, including the vaccine itself, and also implementation, administration and storage costs. To compute the costs of primary care consultations more accurately, we differentiated between primary care consultation at emergency outpatient clinics (EOCs) and general practitioner (GP) offices based on proportions reported by Shin et al. [10].

Health economic outputs were expressed as cost per quality adjusted life year (QALY), cost per case avoided, and cost per severe case avoided. Cost–effectiveness was expressed in terms of an incremental cost–effectiveness ratio (ICER) from healthcare and societal perspectives, and was assessed separately for Rotarix^®^ and RotaTeq^®^. We have used the threshold value of € 73,444 (NOK 657,540) as advised in the Norwegian guidelines for health economic evaluations [33]. This value was adjusted to 2015 price levels by accounting for changes in households’ real disposable income. All costs were converted from Norwegian Kroner to 2015 €, using the average exchange rate during 2015 [1€ = 8.953 NOK] [34]. A yearly discount rate of 4% was applied to costs and health effects, as recommended by the Norwegian guidelines [33].

#### Healthcare costs

To estimate the cost of telephone calls and consultations with GPs, EOCs, and hospital inpatient and outpatient contacts we used the methods and data described by Shin et al. [10], but updated the estimates with Norwegian 2015 diagnosis related group (DRG) prices for hospital–based costs [36], and with GP reimbursement rates adjusted to 2015 price levels using the consumer price index [13].

#### Indirect costs

For children <1 year of age we assumed that no workdays were lost by parents because the parental leave in Norway lasts from 49 to 59 weeks. Ill children aged one year or older were all assumed to require parental care. Based on a recent survey of caregivers of Norwegian children <5 years of age hospitalised with RVGE, the number of workdays lost by caregivers was 3.25 days per child [12]. Based on expert opinion and assumptions in similar studies, the duration of caregiver work loss for non–hospitalized children requiring medical attention was assumed to be 1.5 days, and caregivers of homecare cases were assumed to lose one day [37–39]. A human capital approach was used to value workdays lost, with labour costs computed based on the average full–time equivalent wage of caregivers, added the costs not returned to the worker [13].

#### Vaccination costs

Vaccines included in the national immunisation programme in Norway are procured through the national tender, consequently, the price of programme vaccines is typically somewhat lower than pharmacies’ maximum retail price [40]. Accordingly, the mean baseline vaccine cost per vaccinated child was set to € 54, both for Rotarix^®^ and RotaTeq^®^. The time spent administering the vaccine was based on reports from immunisation nurses (unpublished data), and was higher for RotaTeq^®^ compared with Rotarix^®^ due to an additional dose. Administration time was assumed to decline with the number of doses given, due to parental familiarity with the vaccine. The value of time spent on vaccine administration was estimated based on the average labour cost of public health service nurses, since specific salary data for immunisation nurses were unavailable [13]. The cost of storing the national rotavirus vaccine stockpile was estimated at € 6,953 per year, while the costs of storage at administration venues were not included. We estimated a yearly cost of € 335,083 to account for operational and implementation costs of the vaccination programme. This was assumed to be equal to the budgeted expenses associated with rotavirus vaccination in 2016. Vaccination costs are reported as the cost per vaccinated child, implying two–doses for Rotarix^®^ and three doses for RotaTeq^®^.

#### Quality of life measures

Utility estimates were based on the UK estimates reported by Marlow et al. [35] and these were based on the Health Utilities Index 2 [41] for children, and EQ–5D–5L [42] for parents of inpatients and outpatients [35]. For non–hospitalised cases, we assumed a QALY loss ranging from 0 to the lower confidence interval of reported utility losses for outpatients. Detriments due to mortality were computed based on Norwegian data concerning age–specific losses in expected remaining life years, with a yearly discount rate of 4%.

#### Sensitivity analyses

To account for parameter uncertainty, we conducted Monte Carlo simulations with 10 000 iterations on each parameter using the distributions reported in Table 2. A one–way sensitivity analysis was performed on all key parameters, varying each by ± 20%. Threshold analyses were done for the baseline scenarios to evaluate cost–effectiveness of vaccination at a variety of vaccine prices for Rotarix^®^ and RotaTeq^®^. These analyses were only done from the healthcare perspective.

**Table 2:** Mean parameter values and their sampling distributions for the economic model.

Parameter	Mean	Distribution	Source
**Healthcare costs (€)**			
Inpatient hospital contact	2789	Gamma (0.0003, 7.01)	[10]
Outpatient hospital contact	214	Gamma (0.0031, 5.91)	[10]
GP consultation	24	Gamma (0.0112, 2.43)	[10]
EOC consultation	38	Gamma (0.0061, 2.08)	[10]
**Indirect costs (€)**			
Daily productivity loss caregiver	347	Lognormal (8.04, 0.04)	[13]
Days lost in –and outpatients	3.25	Lognormal (1.17, 0.41)	[12]
Days lost GP and EOC patients	1.5	Lognormal (0.39, 0.18)	Assumption
Days lost homecare cases	1.0	Lognormal (0.00, 0.37)	Assumption
**QALY detriments**			
Inpatients	0.00340	Norm (0.00340, 0.00068)	[35]
Outpatients	0.00290	Norm (0.00290, 0.00058)	[35]
Home and primary care patients	0.00115	Norm (0.00115, 0.00023)	See text
Caregivers of inpatients	0.0082	Norm (0.0082, 0.00164)	[35]
Caregivers of outpatients	0.0046	Norm (0.0046, 0.00092)	[35]
Caregivers of home /primary care patients	0.00145	Norm (0.00145, 0.00029)	See text
Death 0–1 years (discounted)	21.3	Norm (21.3, 4.26)	[9]
Death 1–2 years (discounted)	21.2	Norm (21.2, 4.24)	[9]
Death 2–3 years (discounted)	21.1	Norm (21.1, 4.22)	[9]
Death 3–4 years (discounted)	21.0	Norm (21.0, 4.20)	[9]
Death 4–5 years (discounted)	20.9	Norm (20.9, 4.18)	[9]
**Vaccination costs (€)**			
Administration time Rotarix^®^	20.5	Lognormal (3.0, 0.2)	See text
Administration time RotaTeq^®^	25.0	Lognormal (3.2, 0.2)	See text
Hourly productivity loss administration	424.5	Lognormal (6.05, 0.04)	[13]
Implementation and operational costs	335 083	Norm (335 083, 67 017)	See text

To test the robustness of our findings we conducted a range of scenario analyses to explore the effects of unknown and uncertain parameter values in the model. To simplify comparisons between the different scenarios we report cost–effectiveness in terms of net monetary benefit (NMB), in which costs and effects are combined into a single monetary value using a predefined threshold value (€ 73,444). NMB = (effects x threshold value)–costs. In addition to the baseline scenarios, we evaluated the effect of I) reducing productivity losses due to caregiving from 100% to 50%; II) including parental QALY losses in addition to QALY losses for ill children; III) removing discounting on costs and effects; IV) including the impact of mild adverse events after vaccination by assuming a quality of life loss equal to half of that of mild cases for 1 in 50 vaccinated children; V) assuming vaccine protection to depend on two vaccine doses using the vaccine coverage of the second dose and effective protection in children at four month of age, instead of two months of age in the base case; VI) excluding utility effects from avoided deaths as an attempt to balance out the effect of more severe adverse events such as intussusception. Finally, we assessed cost–effectiveness under best–and worst-case scenarios by conducting multi–way analyses of the best and worst combinations of scenarios I–VI.

## Results

### Epidemiological effects

**Fig 1:**
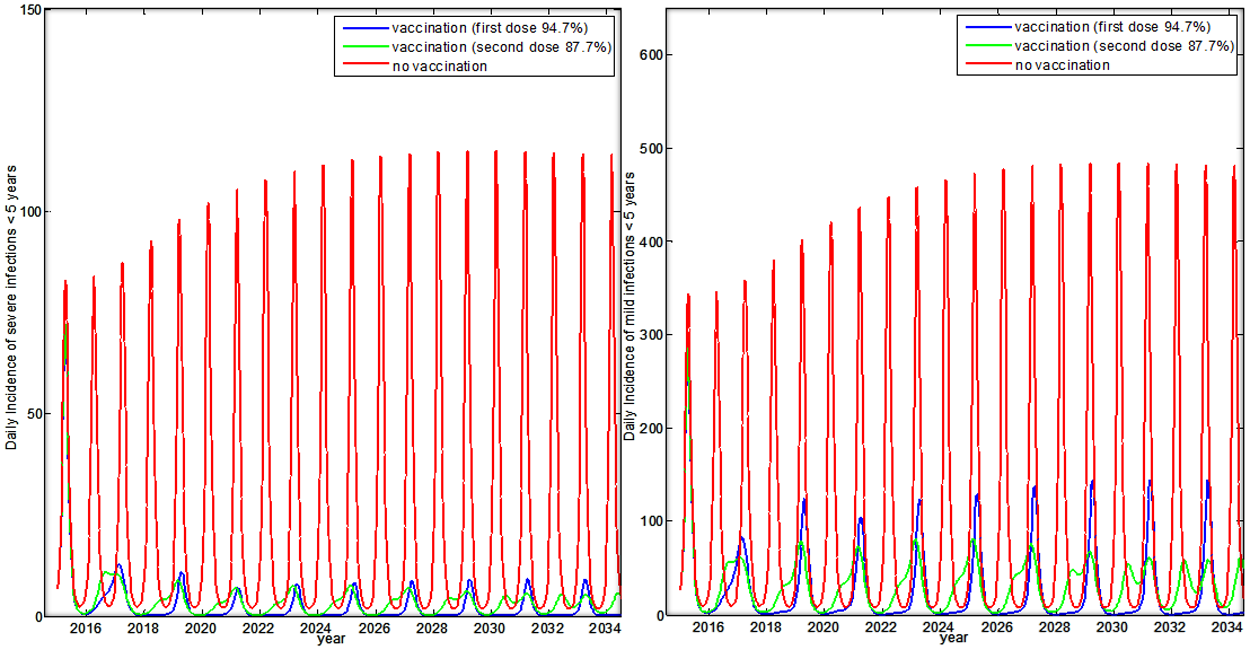
Direct and observed incidence of a) mild and b) severe rotavirus cases with and without a rotavirus vaccination programme between years 2015 and 2034 in Norway.

During the first five years following introduction (2015–2019), the number of hospitalised rotavirus cases and deaths was reduced by 73%, the number of primary care cases was decreased by 70%, and the number of home cases was reduced by 64% (Table 3, Fig 1). The reduction was greatest for cases between one and two years of age, with 45% of severe cases and 32% of mild cases occurring in this age group. (S3 Fig).

**Table 3:** Number of home cases, primary care cases, hospitalised cases and rotavirus deaths predicted by the model, for 2015–2019 and 2016–2019.

Years	Rotavirus cases and deaths	Vaccination Mean (0.20; 0.80)	No vaccination Mean (0.20; 0.80)	Avoided Mean (%)
**2015–2019**	Home cases	64 674 (62 614; 66 726)	179 333 (178 577; 180 088)	114 658 (64%)
	Primary care cases	14 870 (14 550; 15 179)	49 440 (48 682; 50 205)	34 571 (70%)
	Hospitalised cases	2 683 (2 853; 3 222)	10 065 (9 625; 10 468)	7 381 (73%)
	Deaths	0.726 (0; 1)	2.723 (0; 5)	1.996 (73%)
**2016–2019**	Home cases	36 121 (34 074; 38 159)	144 793 (144 183; 145 402)	108 672 (75%)
	Primary care cases	6 856 (6 538; 7163)	39 940 (39 332; 40 558)	33 084 (83%)
	Hospitalised cases	1 078 (1 322; 1 544)	8 144 (7 788; 8 463)	7 066 (87%)
	Deaths	0.290 (0; 0)	2.201 (0; 4)	1.911 (87%)

In 2015, the total number of avoided symptomatic cases (6,302) was lower than for years between 2016 and 2019 wherein the total number of avoided symptomatic cases varied from 24,302–37,327 per year. Reductions in healthcare resource use corresponded with the number of mild and severe cases avoided. The relative reductions in healthcare use was greatest for hospital inpatient and outpatient contacts, while the absolute reduction was greatest for primary care consultations (Table 4).

**Table 4:** Number of healthcare contacts (GP consultations, EOC consultations, hospital inpatient contacts, hospital outpatient contacts) as predicted by the model for 2015–2019 and 2016–2019.

Years	Type of healthcare contact	Vaccination Mean (0.20; 0.80)	No vaccination Mean (0.20; 0.80)	Avoided Mean (% reduction)
**2015–2019**	General Practitioner	10 656 (10 358; 10 946)	35 429 (34 821; 36 034)	24 773 (70%)
	Emergency outpatient clinic	4 215 (4 043; 4 380)	14 013 (13 597; 14 427)	9 798 (70%)
	Outpatient	971 (902; 1039)	3 641 (3 475; 3 807)	2 670 (73%)
	Inpatient	1 712 (1 574; 1 849)	6 423 (6 018; 6 828)	4 711 (73%)
**2016–2019**	General Practitioner	4 913 (4 675; 5 143)	28 621 (28 130; 29 110)	23 708 (83%)
	Emergency outpatient clinic	1 944 (1 832; 2 049)	11 320 (10 984; 11 655)	9 376 (83%)
	Outpatient	391 (350; 430)	2 946 (2 812; 3 080)	2 556 (87%)
	Inpatient	688 (617; 758)	5 197 (4 870; 5 526)	4 509 (87%)

**Fig 2:**
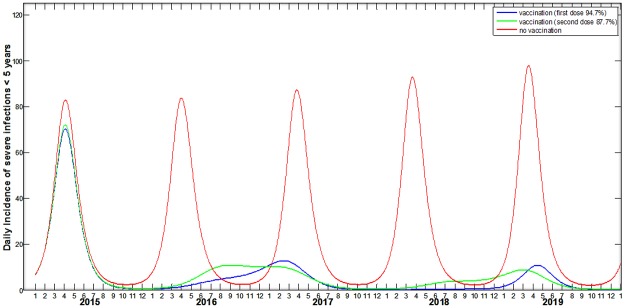
Predicted incidences of severe rotavirus cases in children under five years of age with no vaccination, vaccination coverage for the first dose of 94.75% and vaccination coverage of 87.70% for the second dose.

The model also predicted changes in the rotavirus seasonal pattern post–introduction. The changes included the appearance of biannual peaks in rotavirus incidence, instead of annual peaks, which were present during the pre–vaccine era (Fig 2 and S3 Fig).

## Cost–effectiveness

**Fig 3:**
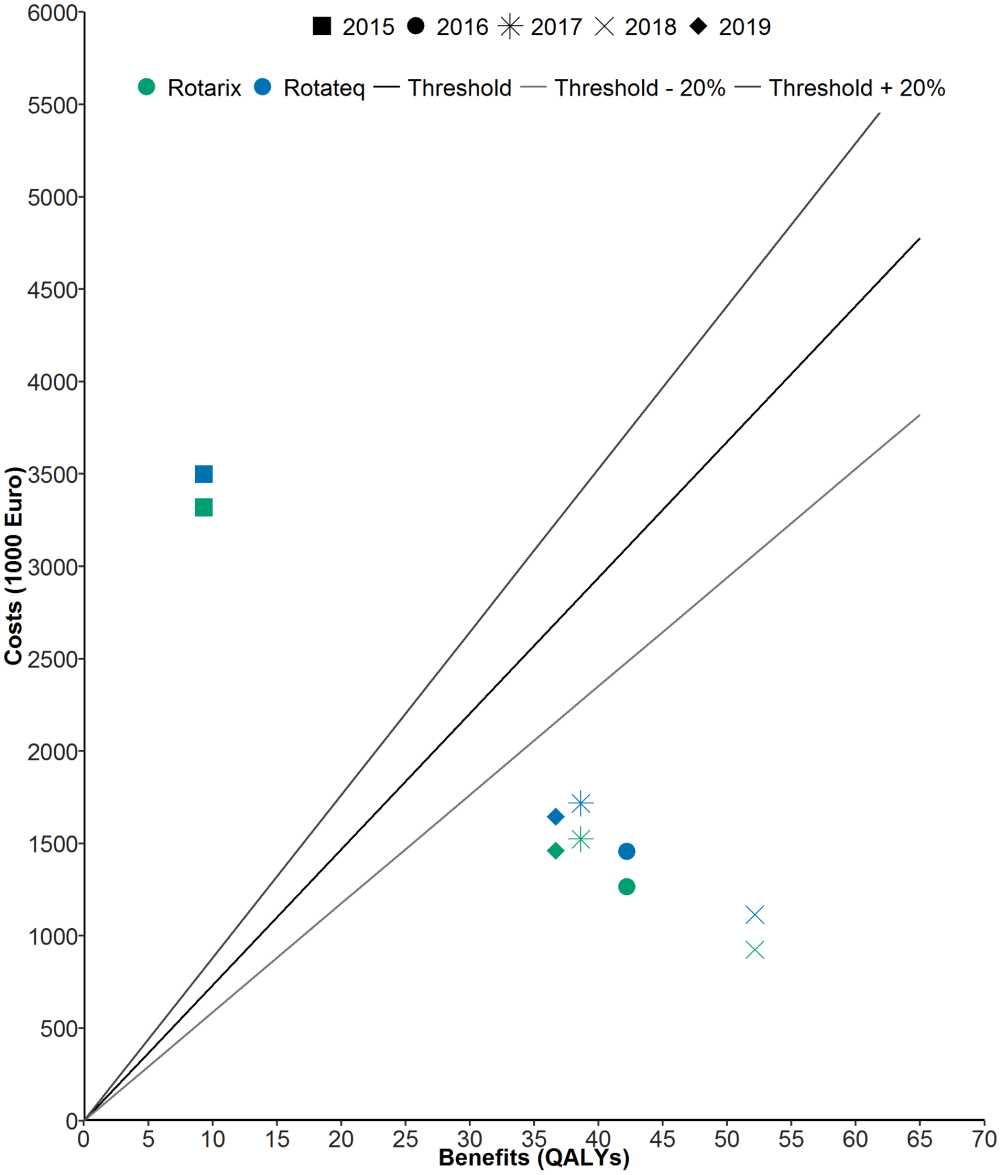
Mean cost–effectiveness under the baseline threshold value of € 73 444 (+/– 20%) of vaccination with Rotarix^®^ (2–doses) and RotaTeq^®^ (three–doses) at 94.7% coverage from a healthcare perspective, Norway, 2015–2019.

In the first five years after introduction, childhood rotavirus vaccination was predicted to be cost–effective from a healthcare perspective and cost–saving from a societal perspective (Table 5). Under baseline vaccines prices, Rotarix^®^ was less costly and thus more cost–effective than RotaTeq^®^. For all years between 2016 and 2019 vaccination was cost-saving from the societal perspective, and cost-effective from the healthcare perspective, but in 2015 vaccination was not cost-effective from the societal or healthcare perspective (Fig 3).

**Table 5:** Cost–effectiveness of childhood rotavirus vaccination, Norway, 2015–2019.

Perspective	Cost–effectiveness outcome	Rotarix^®^ Mean (€)	RotaTeq^®^ Mean (€)
**Healthcare**	Cost per QALY loss avoided	47 447	52 709
	Cost per case avoided	70	77
	Cost per hospitalised case avoided	1150	1278
**Societal**	Savings per QALY loss avoided	187 784	182 552
	Savings per case avoided	275	268
	Savings per hospitalised case avoided	4 553	4 426

During the first five years after vaccine implementation, healthcare costs were reduced by 72%. The absolute cost reduction was greatest for inpatient costs, followed by GP consultations, outpatient hospital contacts and EOC consultations (Table 6). Productivity losses were decreased by 61%, and the largest absolute reductions in productivity losses were a result of fewer homecare cases (Table 6). QALY losses went down by 70%, with the greatest absolute reduction in morbidity (Table 6). Under baseline assumptions, the mean yearly vaccination cost per year for the period 2015–2019, was € 185,624 higher for RotaTeq^®^ compared with Rotarix^®^ (Table 6).

**Table 6:** Costs and quality of life detriments associated with rotavirus vaccination at a coverage of 94.7% during 2015–2019 and without vaccination.

Cost category		Mean (0.20; 0.80) cost without vaccination (€ 1000)	Mean (0.20; 0.80) cost with vaccination (€ 1000)	Avoided Mean (%) (€ 1000)
**Healthcare costs**	GP consultations	822 (456;1 091)	256 (142; 340)	566 (69%)
	EOC consultations	508 (250; 685)	158 (67; 236)	350 (69%)
	Inpatient visits	16 288 (13 171; 18 967)	4 538 (3 645; 5 302)	11 750 (72%)
	Outpatient visits	673 (621; 719)	187 (171; 202)	486 (72%)
**Productivity losses**	Home care	47 805 (34 683; 57 205)	19 696 (14 256; 23 597)	28 109 (59%)
	Primary care	16 793 (14 614; 18 704)	6 091 (5 275; 6 798)	10 702 (64%)
	Hospital care	4 868 (3 348; 5 920)	1 577 (1 078; 1 924)	3 291 (68%)
**QALY detriments**	Morbidity	212 (182; 243)	50 (32; 69)	137 (65%)
	Mortality	57 (0; 116)	15 (0; 22)	43 (75%)
**Vaccination costs**	Rotarix^®^	0 (0; 0)	21 640 (20 996; 22 200)	NA
	RotaTeq^®^	0 (0; 0)	22 580 (21 802; 23 248)	NA

### Sensitivity analyses

#### One–way sensitivity analysis

**Fig 4:**
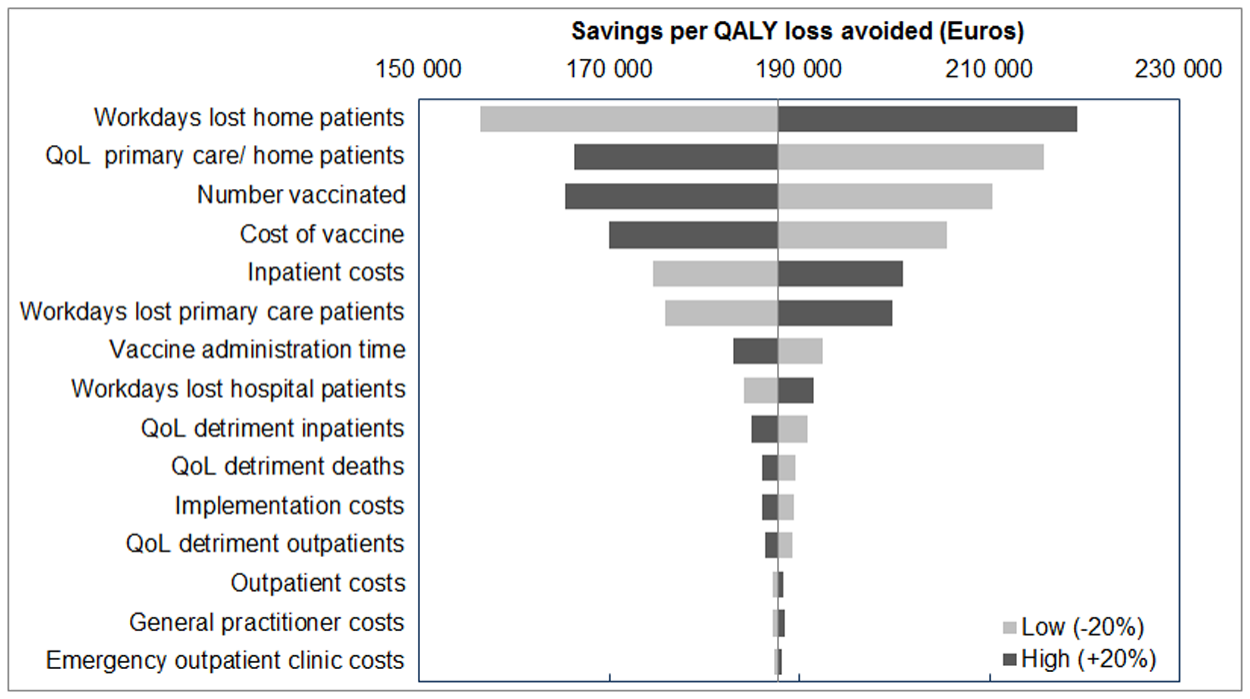
Tornado plot showing impact of varying parameter values by +/–20% on the cost–effectiveness of vaccination with Rotarix^®^ for central cost drivers from a societal perspective, Norway, 2015–2019.

Varying parameters associated with mild rotavirus cases, namely, the health–related quality of life and workdays lost associated with homecare cases had the greatest impact on cost–effectiveness in the one–way sensitivity analyses (Fig 4). In addition, cost–effectiveness of the programme was sensitive to variations in the number of children vaccinated, vaccine price, and inpatient hospital costs (Fig 4).

#### Scenario analyses

**Fig 5:**
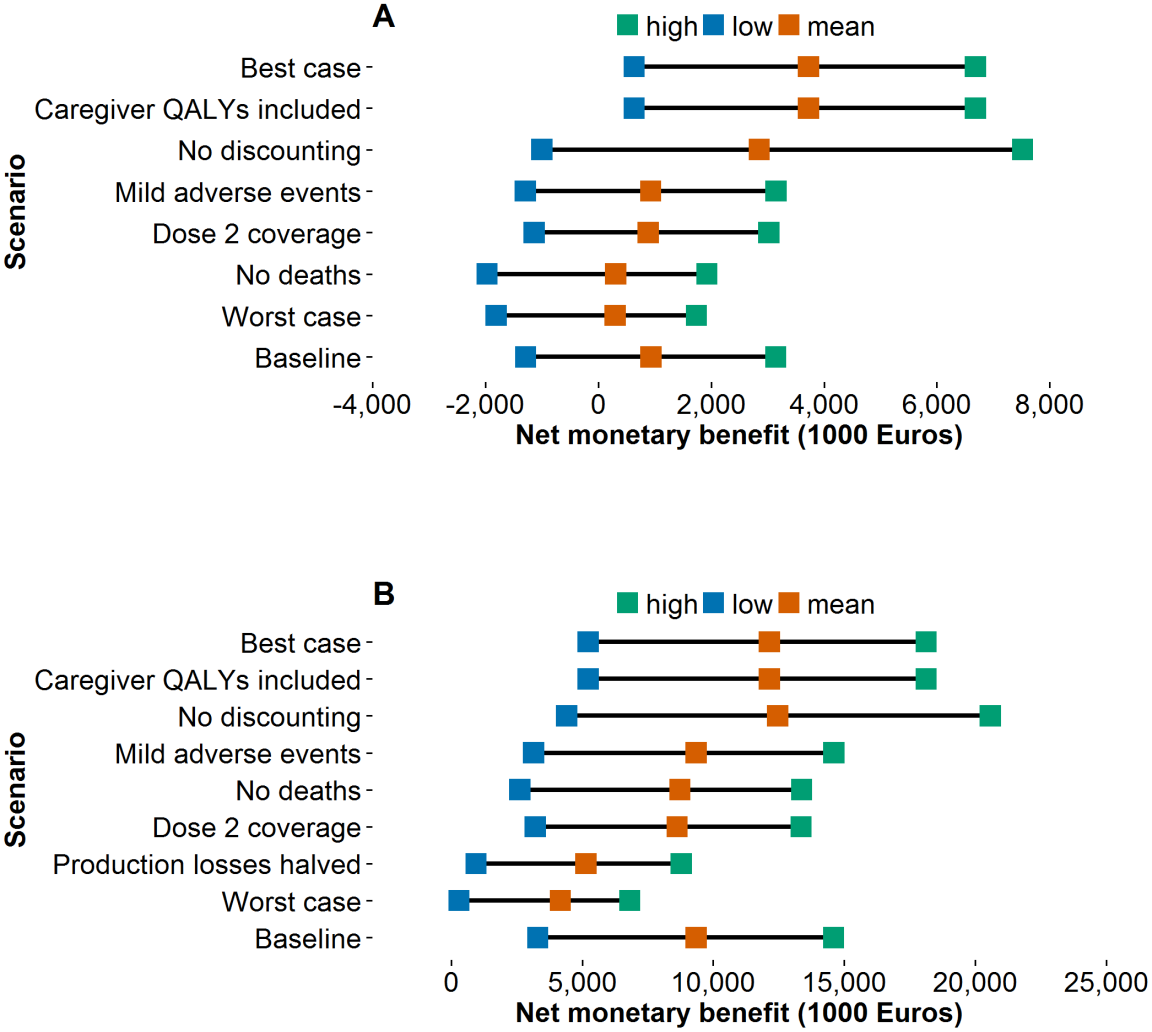
Net monetary benefit under different sensitivity scenarios. Showing the mean, low (0.2 percentile), and high (0.8 percentile) for each scenario with Rotarix^®^ under the (A) Healthcare and (B) Societal perspectives.

In all scenario analyses, vaccination remained a cost–effective strategy (Fig 5). From the healthcare perspective, the best-case scenario resulted in a mean NMB of € 3,728 thousand, while the worst-case scenario resulted in a NMB of € 297 thousand (Fig 5A). In the societal perspective, the best-case scenario resulted in a NMB equal to € 12,148 thousand, and in the worst-case scenario, the NMB was € 4,169 thousand (Fig 5B).

#### Threshold analyses on vaccine prices

**Fig 6:**
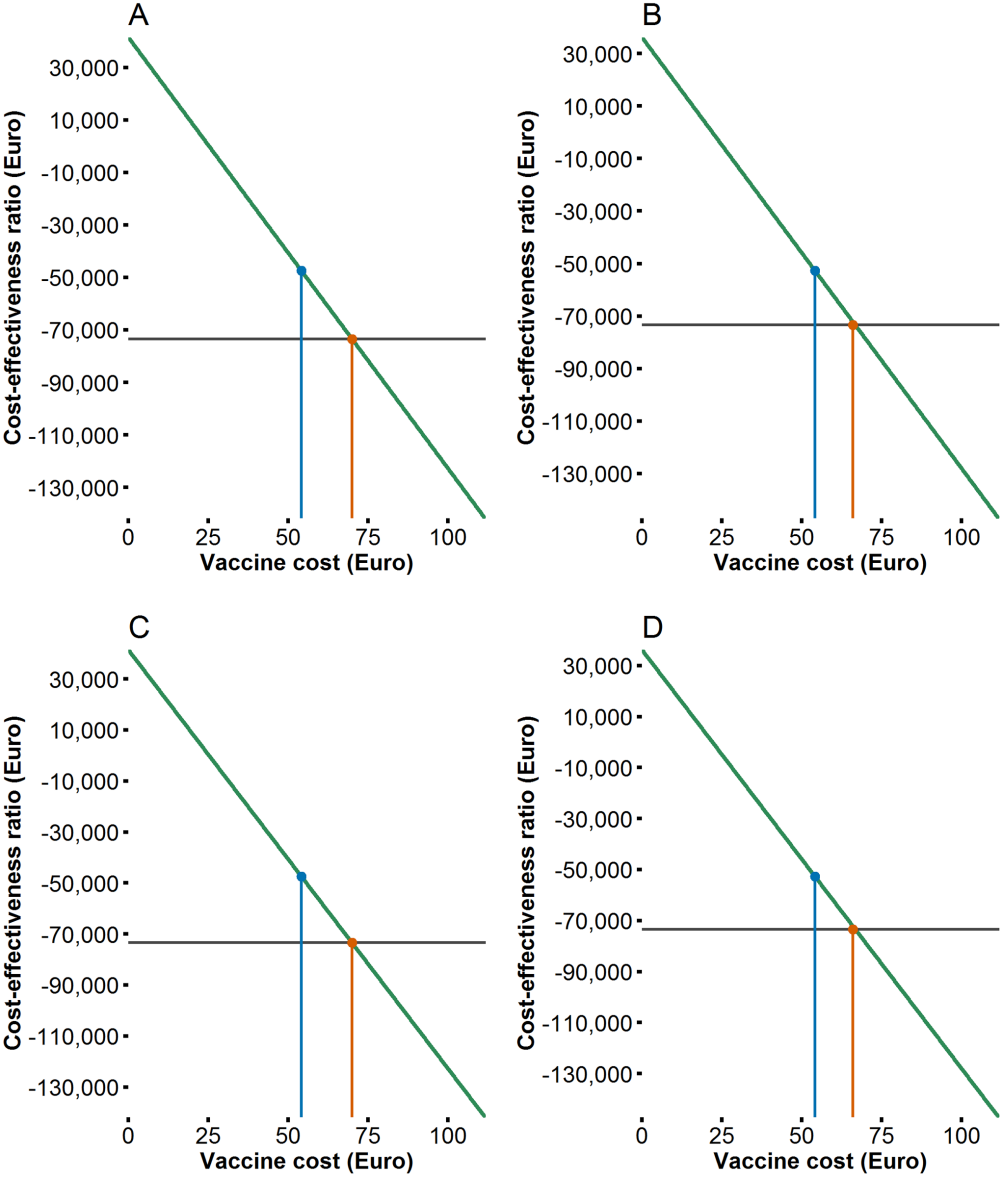
Threshold analysis showing impact of mean vaccine prices per fully vaccinated child on the cost–effectiveness from the healthcare perspective. (A) Rotarix^®^ 2015–2019. (B) RotaTeq^®^ 2015–2019. (C) Rotarix^®^ 2016–2019. (D) RotaTeq^®^ 2016–2019. The blue line represents the average vaccine prices assumed in the model, and the red line represents the break–even price.

Under baseline assumptions, vaccination was cost–saving from the healthcare perspective for vaccine prices of € 25 or lower for Rotarix^®^ and € 22 for RotaTeq^®^. The break–even price per fully vaccinated child for the period 2015–2019 was € 70 for Rotarix^®^ and € 67 for RotaTeq^®^, on average for the period 2015–2019 (Fig 6A and 6B). For the post–introduction period of 2016–2019, the break–even price was € 7.5 higher for both vaccines (Fig 6B and 6D).

#### Acceptability curves

**Fig 7:**
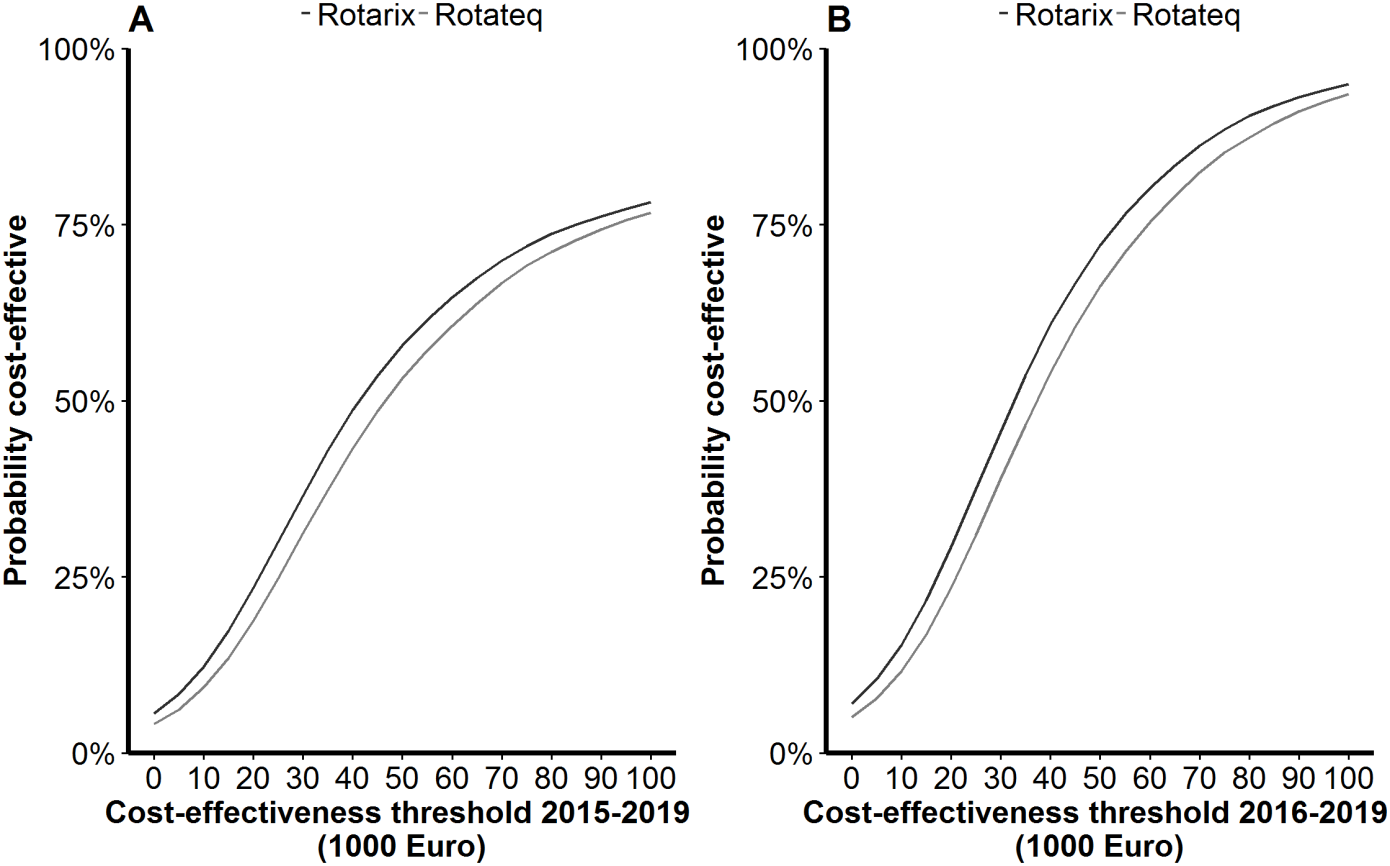
Cost–effectiveness acceptability curves for the healthcare perspective for (A) 2015–2019 and (B) 2016–2019.

During 2015–2019, the likelihood that a rotavirus vaccination programme was cost–effective under baseline assumptions was 71% for Rotarix^®^ and 68% for RotaTeq^®^ from the healthcare perspective (Fig 7A). In the period of 2016–2019 the probability that a vaccination programme was cost–effective from the healthcare perspective was 88% for Rotarix^®^ and 84% for RotaTeq^®^ (Fig 7B).

From 2015–2019, a vaccination programme with Rotarix^®^ was 86% likely to be cost–effective and a programme with RotaTeq^®^ was 85% likely to be cost–effective from the societal perspective. During the post–introduction period, the likelihood of a vaccination programme with Rotarix^®^ and RotaTeq^®^ being cost–effective from the societal perspective was 100%.

## Discussion

The burden of rotavirus vaccination in Norway has been reduced considerably following the implementation of the rotavirus vaccine, and according to our predictions, the burden will remain low during the coming years. Compared with a no vaccination strategy, rotavirus vaccination was predicted to result in a 72% reduction in rotavirus-related costs during 2015–2019. In addition, productivity losses associated with parental caregiving during illness episodes among children were expected to be 61% lower, and QALY detriments due to rotavirus were estimated to decrease by 70%. We found that the ongoing vaccination programme was cost–effective from both a healthcare and a societal perspective. In the baseline scenario, vaccination was cost–saving from a societal perspective for a wide range of vaccine prices, whereas from a healthcare perspective, vaccination was cost-saving for vaccine prices equal to or lower that € 25 and € 22 per fully vaccinated child for Rotarix^®^ and RotaTeq^®^, respectively. Vaccination was also found to be cost–effective from the healthcare and societal perspectives under all the sensitivity analyses considered in our study.

Comparing findings between countries is problematic due to country-specific variations in costs, availability of country-specific data, methodology, and national guidelines for health economic evaluations. Cost–effectiveness of rotavirus vaccination programmes varies between developed countries [6], but also within individual countries [43]. However, our cost per QALY values and break-even vaccine prices are similar to those reported by other countries [6, 43]. In Norway, the threshold willingness-to-pay for a QALY is relatively high (€ 73 444) in comparison with other countries, which results in vaccination being considered cost-effective in our study. Previous studies have used a threshold value of € 30 000 to compare cost-effectiveness across nations; if we apply this threshold value in our study, rotavirus vaccination would be unlikely to be cost-effective (Fig 7), and the break-even price would be lower (Fig 6).

Health economic evaluations of vaccination programmes should ideally be performed prior to vaccine implementation. However, a clear advantage of conducting a post–introductory cost–effectiveness evaluation is access to good–quality data from both before and immediately after implementation. For instance, we based our model estimates of vaccine coverage on real–world observed data as the programme was rolling out. To fit the model, we used data from hospital–based surveillance of acute gastroenteritis cases, which captured changes in the rotavirus incidence in real time and thus allowed timely monitoring of the effects of the rotavirus vaccination. Surveillance data on work absenteeism and healthcare resource use prior to hospital admission were also used to calculate productivity losses and use of healthcare resources in the economic model [12].

Our acceptability curves and a wide range of sensitivity analyses illustrated that rotavirus vaccination in Norway is likely to be cost–effective. Our study should however be interpreted with the following limitations in mind. We conservatively assumed the duration of vaccine–induced protection to be on average two years. Direct vaccine–induced protection may be longer, as suggested by other studies [23, 24]. The duration of natural immunity was assumed to be on average, one year, but this value is in line with the literature [15, 20–22]. The potential economic benefits resulting from herd protection among persons above five years of age [44] were not included in this model, and we may therefore have underestimated the benefits of vaccination. We also lacked Norwegian data on social mixing in the population. Finnish data were used as a substitute, but these data may not be fully representative of the Norwegian population. Our incidence and cost data on hospitalised rotavirus cases were of high quality, but we lacked information about nosocomial rotavirus cases. We could not identify any studies on the burden of nosocomial RVGE cases for the Norwegian setting, and available international studies have been found to report highly variable results depending on the specific methodologies applied [45]. For some model parameters, particularly parameters relating to milder rotavirus cases, we made assumptions based on published data from other settings, and although extensive sensitivity analyses were performed to evaluate the impact of variation in these parameters, this may have impacted the accuracy of our findings. Due to a lack of data we did not include costs of consumables, travel to and from healthcare facilities, or vaccine wastage costs (due to expiry, cold–chain failures, or children regurgitating the vaccine).

Productivity losses associated with parental caregiving of RVGE cases are substantial. In the baseline, we assume zero productivity during the caregiving period. We did not have information about the proportion of parents working part time, or teleworking. To compensate for this we assessed the impact of assuming 50% lower productivity loss during the period in which caregivers were absent from work due to child’s illness. In this scenario, rotavirus vaccination remained cost–saving from the societal perspective, but savings from vaccination were lower. The effect of different concurrent infections among children within a household [46] were not accounted for, therefore the average number of workdays lost may have been slightly overestimated. On the other hand, we may have underestimated the productivity losses because we did not include work loss following the death of a child. While productivity losses following child deaths have been shown to be considerable [47], we were unable to find quantified estimates of workdays lost following the death of a child.

In our study, some of the main uncertainties were a result of limited data to estimate certain parameters. Future studies should take particular care to assess the costs and health consequences among mild rotavirus cases, as these cases account for a considerable proportion of the total rotavirus burden. In addition, once vaccinated cohorts’ age, data should be collected to assess the long-terms effects of rotavirus vaccination; and new health economic evaluations should be conducted.

## Conclusion

Universal childhood rotavirus vaccination in Norway is expected to reduce the rotavirus disease burden considerably, and vaccination can be considered a cost–effective strategy from the societal and healthcare perspectives under the assumed threshold value.

## Supporting information

S1 Fig Weekly cases of children under 5 years of age, admitted due to rotavirus gastroenteritis between 2006 and 2015, reported in sentinel data. (TIF)

S2 Fig Age-specific reduction in the attack rate for mild and severe rotavirus cases between 2015–2019. (TIF)

S3 Fig Daily incidence of A) severe infections and B) mild infections with and without vaccination. (TIF)

S1 Table Results from selected models obtained during fitting procedure using maximum likelihood estimation (MLE). (DOCX)
