# Supplementary figures and images for: Re–evaluation of the cost–effectiveness and effects of childhood rotavirus vaccination in Norway
Source: PLoS One. 2017 Aug 17;12(8):e0183306. doi: 10.1371/journal.pone.0183306 (PMC5560584; doi:10.1371/journal.pone.0183306)

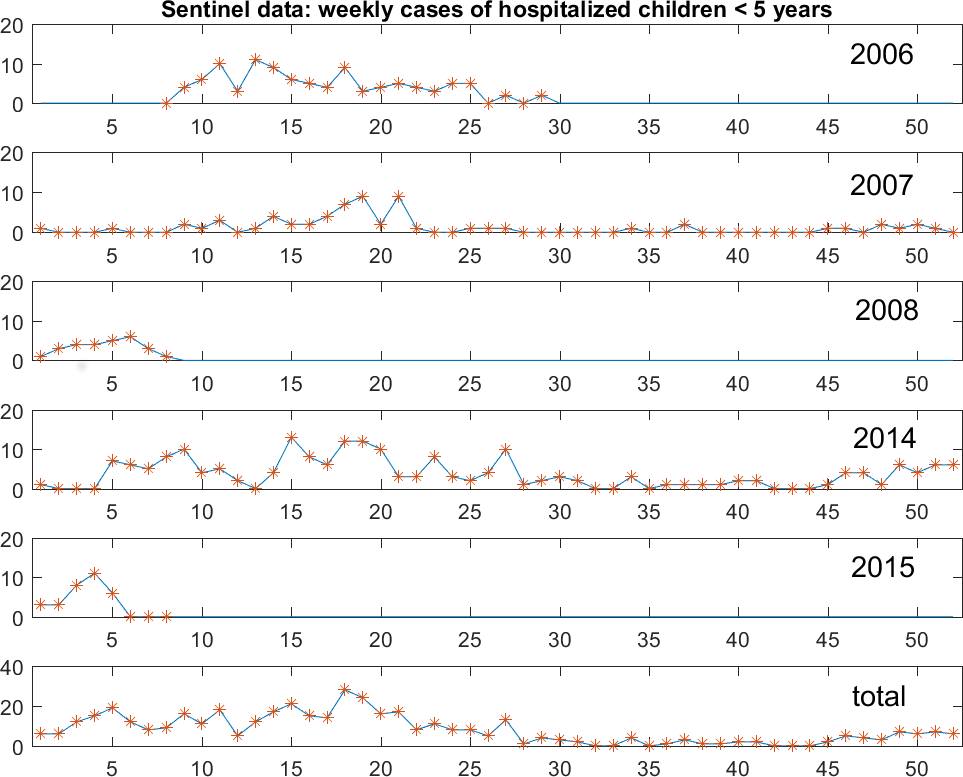

Supplement: S1 Fig — (TIF) [file pone.0183306.s001.tif]

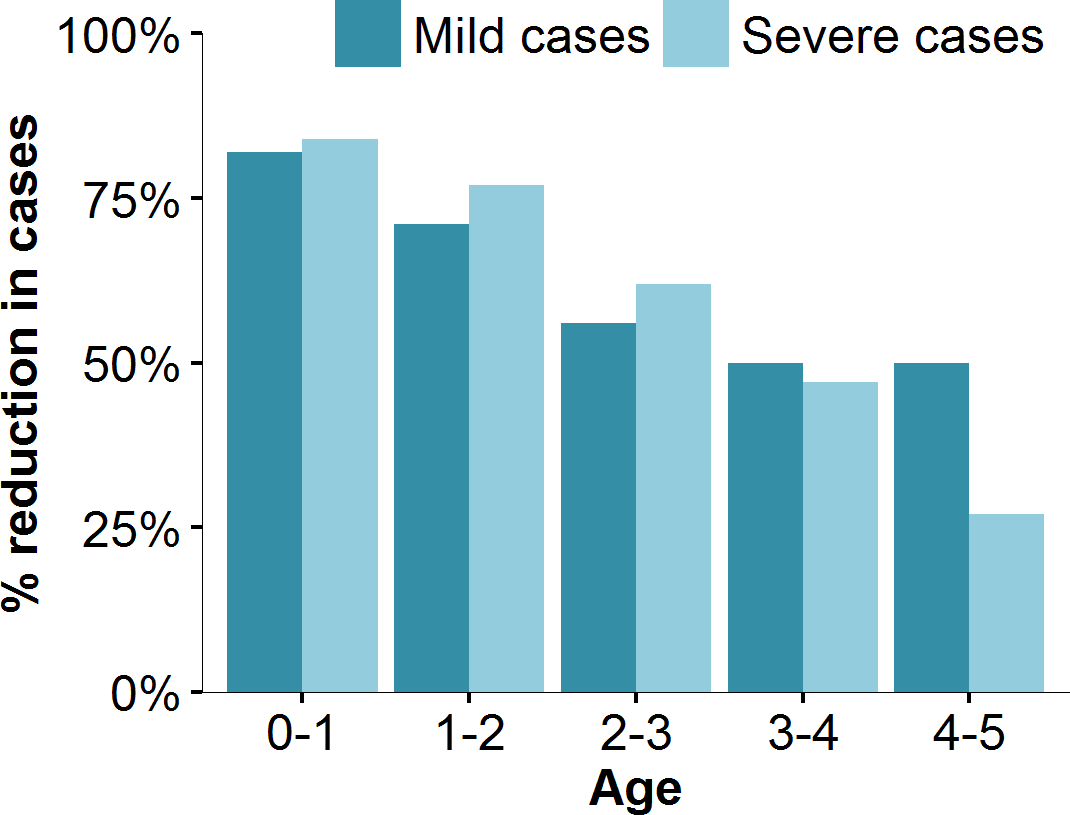

Supplement: S2 Fig — (TIF) [file pone.0183306.s002.tif]

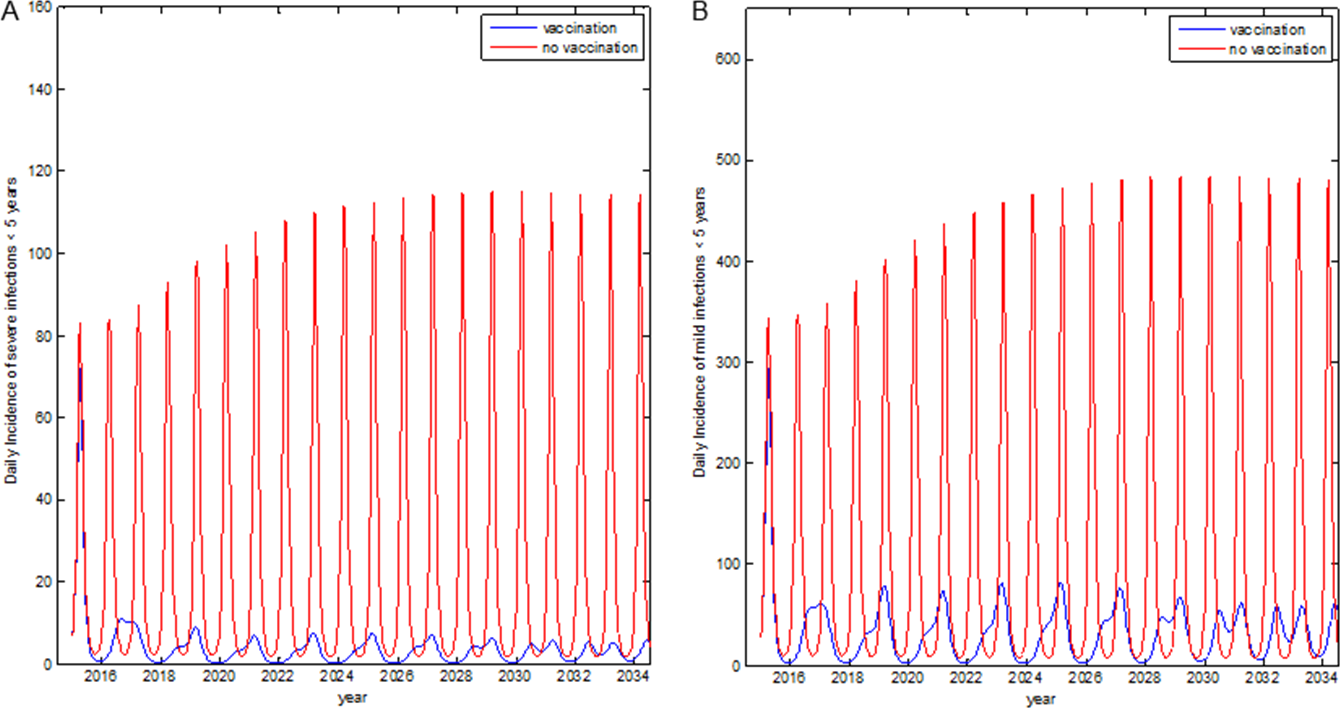

Supplement: S3 Fig — (TIF) [file pone.0183306.s003.tif]
