# Supplementary material for: Re–evaluation of the cost–effectiveness and effects of childhood rotavirus vaccination in Norway
Source: PLoS One. 2017 Aug 17;12(8):e0183306. doi: 10.1371/journal.pone.0183306 (PMC5560584; doi:10.1371/journal.pone.0183306)
Supplement: S1 Table — (DOCX) [file pone.0183306.s004.docx]

**S1 Table: Results from selected models obtained during fitting procedure using Maximum Likelihood Estimation (MLE).**

| **Model** | **Relative contact rate in children < 12 months** | **Relative reporting rate in children > 2.5 years** | **Relative infectivity of 3rd and later infections (kappa)** | **Basic reproductive number of the first infection (R0)** | **Akaike information criterion (AIC)** |
| --- | --- | --- | --- | --- | --- |
| Model 1 | x | x | 0.1 | 12.143 | -46.931 |
| Model 2 | x | x | 0.2 | 11.255 | -46.985 |
| Model 3 | 0.366 | x | 0.1 | 20.675 | -17.053 |
| Model 4 | 0.366 | x | 0.2 | 18.160 | -17.076 |
| Model 5 | x | 0.236 | 0.1 | 5.221 | -21.113 |
| Model 6 | x | 0.236 | 0.2 | 5.221 | -21.174 |
| Model 7 | 0.476 | 0.456 | 0.1 | 13.381 | -10.879 |
| Model 10 | 0.480 | 0.463 | 0.2 | 14.268 | -10.884 |
